# Multi-Organ Morphological Findings in a Humanized Murine Model of Sickle Cell Trait

**DOI:** 10.3390/ijms241310452

**Published:** 2023-06-21

**Authors:** Marcello Trucas, Sabrina Burattini, Susanna Porcu, Michela Simbula, Maria Serafina Ristaldi, Marta Anna Kowalik, Maria Pina Serra, Pietro Gobbi, Michela Battistelli, Andrea Perra, Marina Quartu

**Affiliations:** 1 Department of Biomedical Sciences, Section of Cytomorphology, University of Cagliari, Cittadella Universitaria di Monserrato, 09042 Monserrato, Italy; marcello.trucas@unica.it (M.T.); mpserra@unica.it (M.P.S.); quartu@unica.it (M.Q.); 2 Department of Biomolecular Sciences, Campus Scientifico “Enrico Mattei”, University of Urbino Carlo Bo, Via Ca’ le Suore 2—Località Crocicchia, 61029 Urbino, Italy; sabrina.burattini@uniurb.it (S.B.); pietro.gobbi@uniurb.it (P.G.); 3 Italian National Research Council (CNR)—IRGB, Cittadella Universitaria Monserrato, 09042 Monserrato, Italy; susanna.porcu@irgb.cnr.it (S.P.); michela.simbula@irgb.cnr.it (M.S.); mariaserafina.ristaldi@irgb.cnr.it (M.S.R.); 4 Department of Biomedical Sciences, Unit of Oncology and Molecular Pathology, University of Cagliari, Cittadella Universitaria di Monserrato, 09042 Monserrato, Italy; ma.kowalik@unica.it (M.A.K.); andrea.perra@unica.it (A.P.)

**Keywords:** sickle cell disease, sickle cell trait, morphology, ultrastructure, hematology, transmission electron microscopy, humanized model, Townes mouse, anatomy

## Abstract

Sickle cell disease (SCD) is caused by the homozygous beta-globin gene mutation that can lead to ischemic multi-organ damage and consequently reduce life expectancy. On the other hand, sickle cell trait (SCT), the heterozygous beta-globin gene mutation, is still considered a benign condition. Although the mechanisms are not well understood, clinical evidence has recently shown that specific pathological symptoms can also be recognized in SCT carriers. So far, there are still scant data regarding the morphological modifications referable to possible multi-organ damage in the SCT condition. Therefore, after genotypic and hematological characterization, by conventional light microscopy and transmission electron microscopy (TEM), we investigated the presence of tissue alterations in 13 heterozygous Townes mice, one of the best-known animal models that, up to now, was used only for the study of the homozygous condition. We found that endothelial alterations, as among which the thickening of vessel basal lamina, are ubiquitous in the lung, liver, kidney, and spleen of SCT carrier mice. The lung shows the most significant alterations, with a distortion of the general tissue architecture, while the heart is the least affected. Collectively, our findings contribute novel data to the histopathological modifications at microscopic and ultrastructural levels, underlying the heterozygous beta-globin gene mutation, and indicate the translational suitability of the Townes model to characterize the features of multiple organ involvement in the SCT carriers.

## 1. Introduction

Sickle cell disease (SCD) is a hemoglobinopathy caused by a single nucleotide mutation in the hemoglobin (Hb) beta chain. The change in a single amino acid, valine instead of glutamic acid (p.Glu7Val), leads to structurally altered hemoglobin; named hemoglobin S (HBS), that, unlike normal hemoglobin (HBA); undergoes polymerization when exposed to challenging conditions (reduction in plasma pH, low oxygen saturation, dehydration, and low temperature) [1,2]. This change causes erythrocyte (red blood cells—RBCs) alterations in shape (sickle cells), stiffness, and reduction in function [3,4]. Altered erythrocytes tend to damage the endothelium and aggregate, hindering regular blood flow [5]. The resulting syndrome is related to the severity of diffuse microvascular occlusion. Homozygous subjects (HBB^s/s^, SCD-affected) develop acute and chronic ischemic disease that can affect the lung, kidney, liver, spleen, heart, skin, and joints [6,7]. On the other hand, heterozygous subjects (HBB^A/S^ carriers of sickle cell trait—SCT) are virtually considered healthy subjects [8,9,10]. However, new clinical evidence is leading to a reconsideration of the importance and frequency of some complications observed in SCT carriers: exertion-related injury, chronic kidney disease (CKD), renal medullary carcinoma, thrombo-embolic diseases, and arteriopathies [11,12,13,14,15].

There have been several screening campaigns to identify the carriers of the trait, mostly among newborns and some categories of workers [16,17]. However, since SCT carriers lead a normal life and are predominantly asymptomatic, data regarding the possible presence of tissue alterations in heterozygous patients are scanty [18,19,20].

In this context, animal models are crucial to defining the morphological, molecular, and biochemical features of this hemoglobinopathy [19,20,21]. The first model of sickle cell disease was the SAD mouse (β^6^Val substitution of the βS chain, as well as two other mutations, S-Antilles-D Punjab) [22], exhibiting a mild/moderate disease with sickle cells containing Hb polymers in vivo, but no anemia in adulthood. However, in conditions of deoxygenation, this model shows histological signs of SCD and, for this reason, was extensively used to evaluate potential therapeutic agents against the abnormal potassium efflux and dehydration of sickle erythrocytes in the homozygous human form. In fact, this type of model contains the so-called “super sickling” hemoglobin that polymerizes very easily [23].

Subsequently, the development of anemic but viable mouse models of sickle cell anemia lose speed in the face of two main problems, i.e., the replacement of the mouse hemoglobin with human HbS and the prevention of fetal death due to a high HbS expression [22]. Thus, the design of the “HbS-only” models, such as the Townes and the Berkeley, came as a promising avenue for the understanding of phenotypes with different degrees of severity [24]. However, while all the available models are specifically designed to analyze the SCD clinical implications, the SCT condition has been neglected on the assumption that it shows no histopathological alterations.

To date, the chimera mouse developed by T.M. Townes, expressing human HbS, is one of the most used animal models of SCD [25,26,27]. In this model, the murine adult α-globin and β-globin genes were replaced, respectively, with the human α-globin and the human sickle β^S^- genes. While the homozygous Townes mice are notoriously affected by SCD and have been well characterized, heterozygous “sickle-cell trait” mice, are not commonly used as a model of SCT [28,29]. It is well known that, in homozygous Townes mice, histopathological and hematological findings are the same that we could observe in the more severe human forms of SCD [30]; nevertheless, there is a lack of systematic and multi-organ data on the microanatomical alterations of SCT carrier mice.

A few clinical data reporting evidence for organ dysfunction in heterozygous patients [31] highlight the need to investigate whether the hematological and clinical alterations are reflected by matching tissue alterations in SCT carriers. Therefore, using heterozygous Townes mice, this pilot study aims to contribute to the knowledge of the morphological features displayed by the SCT carrier mice through multi-organ histological and ultrastructural analyses.

## 2. Results

### 2.1. Hematological Values

The mean corpuscular hemoglobin (MCH) of SCT mice (*HBB^A/S^*) showed a significant reduction when compared to controls *(HBB^A/A^*). By contrast, red blood cell count (RBC), hemoglobin (Hb), hematocrit (Hct), and mean corpuscular Hb concentration (MCHC) values were similar in the two groups. When compared with *HBB^S/S^*, these hematological values, except for the hematocrit, are significantly higher in *HBB^A/S^* (Table 1).

### 2.2. Erythropoiesis, Spleen Size, and Organ Iron Content

In adult *HBB^A/S^* mice, no patently statistically significant differences in the level of erythropoiesis in bone marrow cells, spleen size, and organ iron content were detected when compared to the adult *HBB^A/A^* phenotype.

The spleen index value was significantly higher in *HBB^S/S^* mice compared to control *HBB^A/A^* mice. In *HBB^S/S^* mice the iron concentration assayed in the liver, spleen, and heart showed a significant iron overload in the liver and spleen compared to control *HBB^A/S^* mice. As for the heart, the scored values did not reach statistical significance (Table 2).

### 2.3. Histopathology

**Figure 1:**
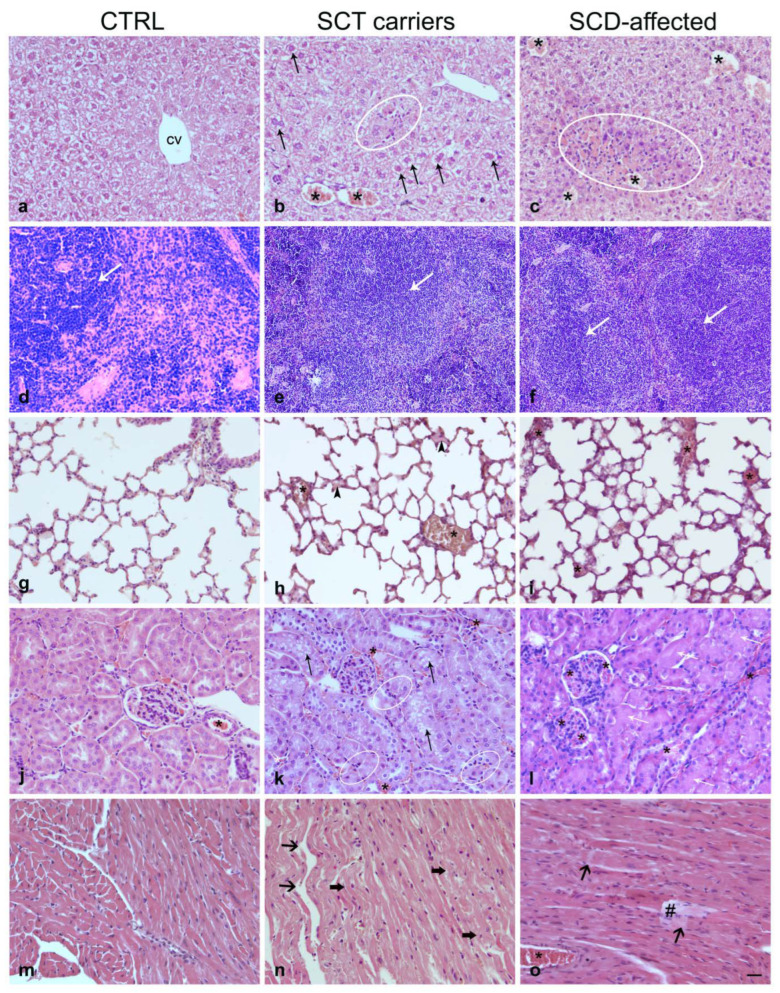
Histopathology of the liver (**a**–**c**), spleen (**d**–**f**), lung (**g**–**i**), kidney (**j**–**l**), heart (**m**–**o**) in controls, SCT carriers, and SCD affected mice. (**a**–**c**) show hepatocyte vacuolization (arrows), congested vessels (asterisks), and coagulative necrosis with mononuclear infiltrates (oval shapes). (**d**–**f**) show the progressive increase in the lienal white pulp (arrows) in SCT and SCD animals. (**g**–**f**) illustrate the pulmonary parenchyma with aspects of edema (arrowheads) and congested vessels (asterisks). (**j**–**l**) the renal cortex shows congested vessels (asterisks), tubular cell vacuolization (black arrows), and swelling (white arrows). (**m**–**o**) show the waving (solid arrows), fragmentation (wide arrows), and pale staining (#) of the myocardiocytes. Scale bar A-N=O = 50 µm.

Compared to controls, the SCT carrier mice showed histopathological features (Figure 1b,e,h,k,n) that were classified according to an SCT carrier lesion grading, simplified from Manci et al. [21]. We included three degrees of injury: normal/minimal, mild/moderate, and severe. Most examined organs from SCT carrier mice showed congested small blood vessels compared to those of control preparations, consistent with minimally impaired blood flow; by contrast, the lungs showed white blood cell infiltration, congested vessels, and diffuse edema, suggestive of ongoing inflammatory changes (Figure 1; Table 3). The SCD-affected mice presented evident and widespread ischemic signs in all analyzed organs (Figure 1c,f,i,l,o). The following detailed description of organ lesions is focused on the SCT carriers.

#### 2.3.1. Liver

Although the hepatic tissue architecture was preserved, a minimal hepatocyte vacuolization and small areas of coagulative necrosis and inflammation were detectable (Figure 1a–c).

#### 2.3.2. Spleen

The lienal parenchyma showed a few areas of vascular ectasia and increased white pulp (Figure 1d–f). No infarcts were present in any of the samples examined.

#### 2.3.3. Lung

A minimal degree of organ lesions was observed in the lungs where minimal capillary congestion occurred. A focal alveolar edema could also be observed. Evaluation of semithin sections showed a thicker alveolar basement membrane than controls, giving the parenchyma a closely packed and irregular appearance (Figure 1g–i).

#### 2.3.4. Kidney

In the kidney, slight vessel congestion and swelling of some tubular cells were observed (Figure 1j–l).

#### 2.3.5. Heart

Samples from SCT carriers showed morphology similar to controls (Figure 1m–o). The myocardiocytes were normally stained, with clearly visible nuclei; no ischemic areas or collagen fiber deposits were present. Rare focal weaving and fragmentation could also be observed.

### 2.4. Transmission Electron Microscopy

**Figure 2:**
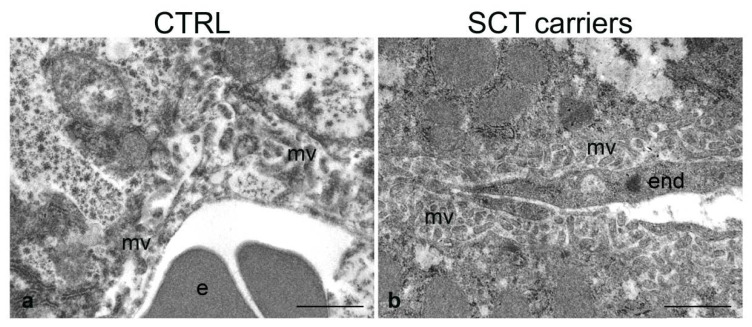
Ultrastructure of the liver in normal (CTRL) and SCT carrier mice. Sinusoidal capillaries with hepatocyte microvilli (mv) projecting into the Disse space. e, erythrocyte; end, endothelial cell. Scale bars: (**a**,**b**), 1 µm.

**Figure 3:**
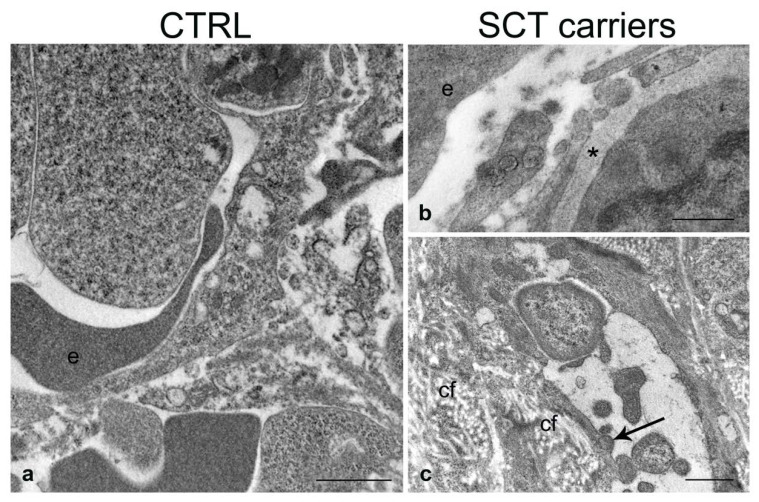
Ultrastructure of the spleen in normal (CTRL) and SCT carrier mice. Vessels in the white pulp; (**b**,**c**) endothelial cells showing enlarged basal membrane (asterisk) and cytoplasmic blebs along the endothelial lining (arrow). cf, collagen fibers; e, erythrocyte. Scale bars: (**a**,**c**), 1 µm; (**b**), 0.5 µm.

**Figure 4:**
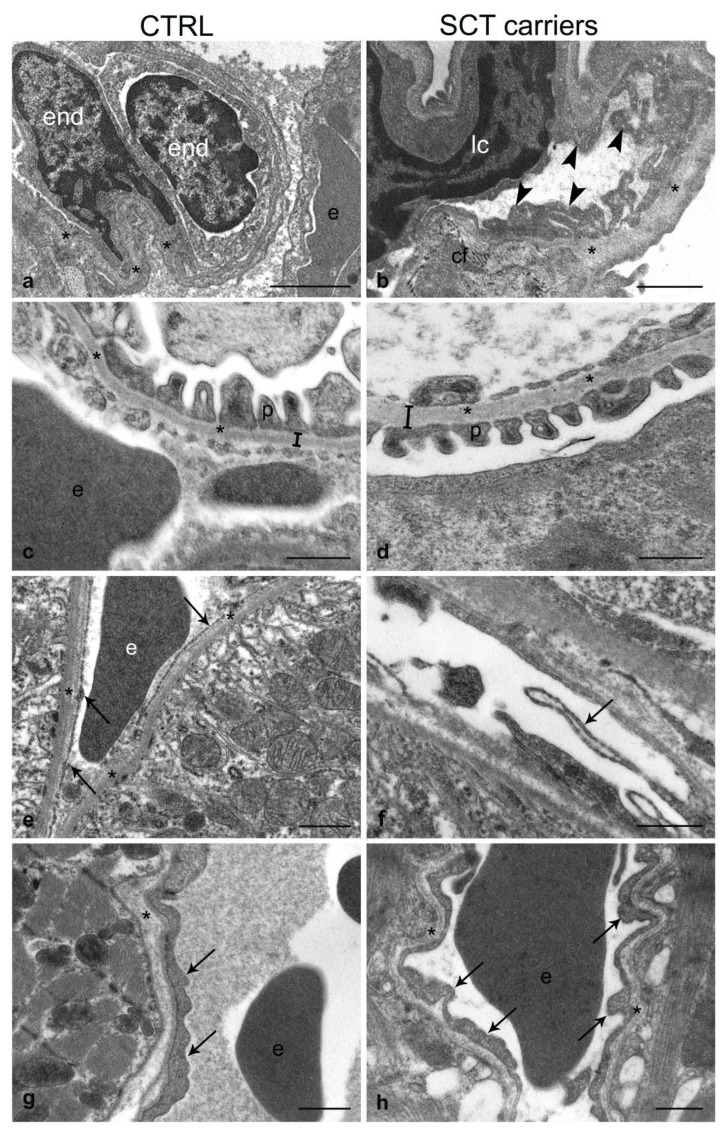
Ultrastructure of the lung (**a**,**b**), kidney (**c**–**f**), and heart (**g**,**h**) in normal (CTRL) and SCT carrier mice. (**a**,**b**) Pulmonary vessels; the basement membrane (asterisks) and endothelial pinocytotic vesicles (arrowheads) are shown in the SCT carriers. (**c**,**d**): renal corpuscle; vertical bars denote the thickness of the basement membrane (asterisks) in CTRL vs. SCT carriers. (**e**, **f**) renal peritubular vessels; arrows in (**f**) point to luminal extroflexions of the endothelial lining. (**g**,**h**) cardiac tissue; shown are the basal membrane (asterisks) and luminal extroflexions of the endothelial lining (arrows). cf, collagen fibers; e, erythrocyte; end, endothelial cell; lc, lymphocyte cell; p, podocyte processes. Scale bars: (**a**), 2 µm; (**b**–**g**), 1 µm; (**c**,**g**,**h**) 0.5 µm.

The ultrastructural analysis revealed that the SCT carrier mice showed some characteristic alterations that are enlisted below (Figure 2, Figure 3 and Figure 4).

#### 2.4.1. Liver

Control mice (C1, C2) showed normal tissue architecture and normal endothelium of the hepatic intralobular capillaries (Figure 2a). By contrast, SCT carrier mice (M1, M2) showed endothelial cell and hepatocyte alterations compared to controls (Figure 2b). Thus, M1 showed an evident thickening of the endothelial lining vs. controls, while M2 showed intermediate morphological signs, with varying endothelial changes. As for the hepatocytes; in M1 and, to a lesser extent, M2 tissue samples; they showed increased microvilli that appeared thickened and packed throughout the entire perisinusoidal Disse’s space.

#### 2.4.2. Spleen

Control mice showed normal spleen tissue architecture and regular vessel structure, in terms of endothelial luminal surface and basement membrane (Figure 3a). In the SCT carrier mice, the vessels, studied mostly in the marginal zone due to its better preservation in semithin sections, revealed a patent enlargement of the endothelial basement membrane (Figure 3b,c). Occasionally, cytoplasmic blebs protruding in the vessel lumen could be observed. Collagen fiber deposits, suggestive of ongoing fibrosis and possible tissue infarcts, were evident in the tissue areas adjacent to the thickening of the basement membrane.

#### 2.4.3. Lung

The pulmonary capillaries of SCT carrier mice showed irregular morphology due to numerous extroflexions of the endothelial luminal surface and an evident thickening of the basement membrane compared to controls (Figure 4a,b). Additionally, erythrocytes with altered shape and deposition of collagen fibers at the alveolar–capillary interface were also observed.

#### 2.4.4. Kidney

The kidney of control mice exhibited regular tissue architecture in both the glomerular and tubular districts (Figure 4c,e). By contrast, the kidney of SCT carrier mice showed glomerular basement membrane thicker than controls and endothelial detachment from the basement membrane at the level of the peritubular capillaries (Figure 4d,f). Moreover, the endothelium of SCT carriers presented “rosary chain” bulges (Figure 4f).

#### 2.4.5. Heart

When compared to controls, the myocardial ultrastructure of the SCT carrier mice revealed slight abnormalities of the vessels and myocardiocytes (Figure 4g,h). Thus, the endothelial cells presented several luminal extroflexions appearing wider than those observed in the controls, while the adjoining myocardiocytes were vacuolated and showed a patent increase in the number of pinocytotic vesicles.

## 3. Discussion

To the best of our knowledge, this study addresses for the first time the issue of a systematic analysis of SCT carrier-related organ modifications at the histological and ultrastructural level using the validated model of the Townes mice. Our primary outcome is that the heterozygous mice; even in the absence of patent hematological differences compared to the controls; carry characteristic lesions of the tunica intima of small vessels, that are especially found in the liver, spleen, kidneys, and lungs. While anomalies of the endothelial lining associated with SCD-affected patients and mouse models have been previously reported [32,33,34], the evidence of their association with the SCT carrier condition is still fragmentary [35,36]. The histopathological differences among the SCD-affected, the SCT carrier, and control mice have been addressed in very few studies. Thus, Nguyen et al. [30] showed that, in the Townes model, the SCD-affected mice have increased erythropoiesis, hepatic necrosis, intrahepatic hemosiderin deposits, and some small renal glomeruli lesions. They also observed a marked increase in the spleen volume, probably due to the conditions of severe congestion and increased erythropoiesis. By contrast, no organ lesions were detected in the SCT carrier mice of the same study, which appeared essentially normal [30]. Another study carried out on a different model of SCD, the Berkeley’s mouse; addressed mainly the topic of vascular congestion in basal conditions and after hypoxia and reoxygenation [37]; and demonstrated normal morphology of the liver, lung, kidney, and spleen, except negligible vascular congestion in the SCT mouse.

Our results, while in the context of a pilot study, given the low number of animals we could obtain for the ultrastructural analysis, suggest an association between the SCT carrier condition and abnormalities of the small vessels’ tunica intima, the worst impairment occurring in the endothelium and subendothelium of the lung vessels, with an enlargement of the vascular basal lamina. In our hands, the alveolar–capillary interface appeared morphologically altered by the interposition of collagen fibers and irregularly shaped erythrocytes stacked into the capillaries, therefore suggesting the possibility of slowed blood flow. While the lung diseases in SCD-affected subjects are well known [38,39], the SCT carriers do not present clinically significant pulmonary alterations [27,34,40,41]. Our observations in the lungs of SCT carrier mice may thus represent a sign of early organ damage whose morphological and functional evidence may increase in the long term and under recurrent conditions of hypoxia and pH reduction.

Kidney structural and functional abnormalities are well known in SCD-affected subjects, although some pathophysiological aspects have yet to be clarified [42]. Regarding the histopathology of the kidney of the SCT carrier mouse, our findings suggest that the thickening of the vascular basement membrane may represent a prodrome of chronic damage, possibly due to a decreased glomerular ultrafiltrate and dysregulation of tubular reabsorption. On the other hand, the observed detachment of the endothelial lining of the peritubular capillaries from the basal membrane might be interpreted as an early sign of peritubular capillary rarefaction (PTC). The PTC is a prominent histological characteristic of CKD, correlates with impaired kidney function, and is predictive of end-stage renal failure in patients and mouse models with CKD [43,44,45]. Interestingly, the decrease in endothelial fenestrations and the thickening of the tunica intima have been indicated as a sign of PTC in both the human kidney and the mouse CKD model [43].

The adhesion of sickle red blood cells (RBCs) to the endothelium-associated proteins is thought to contribute to the vascular occlusion crisis in SCD. In the case of the sickle RBC, it is known that the laminin α5 chain is one of the molecules that mediate their adhesion to the extracellular matrix [46]. On the other hand, studies carried out on animal models of diabetes, aging, and renal neoplasia show that the enlargement of the basement membrane may be related to an increase in the glucidic component rather than to collagen IV [47], and that laminin glycation decreases cell adhesion [48,49,50]. Thus, considering these findings, it could be hypothesized that, in SCT carriers, the increase in laminin glycation may represent the means through which the endothelial lining may react and decrease the adhesiveness of the basement membrane when exposed to the blood shear.

Our study shows that the thickening of the endothelial lining is also a hallmark of the liver of SCT carrier mice, suggesting that it may represent the morphological expression of an adaptive response to the persistent contact with the stiff sickle RBCs which, by preventing a regular blood flow [51], stimulate hepatocyte plasticity to ensure the efficiency of plasma exchanges. While it cannot be excluded that the increased microvilli may represent the hepatocyte response to the decrease in endothelial fenestrations [43], it is interesting that the correlation between the number of hepatocyte microvilli and the level of endothelial activation of the hepatic sinusoids has been reported in mice and humans [52,53].

As regards the slight fibrosis we observed in the spleen of SCT carrier mice, even in the absence of splenomegaly or iron accumulation, it may be interpreted as an accompanying feature of the possibly increased spleen workload in the elimination of damaged erythrocytes [54]. As for the pronounced endothelial plasma membrane luminal extroflexions observed in our preparations, it is tempting to suggest that they support the notion that circulating microvesicles may even form in SCT carriers [55,56]. Thus, it would come as no surprise if these extroflexions, often containing mitochondria, could detach and become circulating microparticles. Indeed, the presence of extracellular circulating mitochondria from different cell types, under baseline and pathological conditions, has already been described in both patient and mouse models as a physiological response to cellular stress [57,58].

According to clinical evidence showing no association between SCT and the risk of developing heart disease [59,60], in our hands, the heart of SCT carrier mice has proven to be unaffected by microlesions at both light and ultrastructural levels. Nonetheless, the presence of endothelial luminal extroflexions resembles the morphological features observed in the spleen and shares similar characteristics to the human umbilical vein of SCT carrier mothers where the endothelial lining also showed an extended luminal profile with budding plasmalemmal vesicles [18].

Concerning the hematological aspects, it is worth mentioning that Townes SCD mice have both human α- and β-globin genes knocked into the mouse locus and produce only human hemoglobin in fetal and adult definitive erythropoiesis. When carrying two copies of the βS allele, Townes mice exhibit many of the hematological features of SCD, including anemia, short RBC half-life, RBC sickling, and high peripheral blood reticulocyte frequencies. In *HBB^S/S^* mice, the erythropoiesis, estimated by the ratio between early precursor (Ter119^+^ CD71^+^) and more mature bone marrow-derived erythroid cells (Ter119^+^ CD71^−^), is to some degree ineffective. This issue also emerges when compared to SCT mice that, in the context of our study, exhibit an intermediate phenotype even for hematological values (see Table 1 and Table 2), according to the moderate histological lesions (see Figure 1). Splenomegaly, due to stress erythropoiesis and red cell sequestration, is another characteristic disease symptom seen in SCD patients and is one of the hallmarks of the Townes SCD model [2]. Severe chronic hemolytic anemia is associated with iron overload and in *HBB^S/S^* mice the iron concentration showed a significant iron overload in the liver and spleen compared to *HBB^A/A^* mice [2], and to *HBB^A/S^* (see Table 2). In the heart, likely due to the sample variability, no differences in iron accumulation were observable in homozygous compared to heterozygous mice.

So far, the available preclinical and clinical studies [46,47,48,49] indicate that the condition of the SCT carrier, even if insufficient to elicit symptoms as evident as those caused by SCD, might bear some characteristic morphological alterations that affect the organ homeostasis. Despite the relatively small sample size and the lack of metabolic parameters of SCT carrier mice, we believe that our findings may pave the ground for further insights aimed at clarifying the molecular regulation of the endothelial response in SCT carriers. Furthermore, our results underpin the utility of the SCT carrier animal model for the study of the mechanisms by which vascular complications may occur in previously asymptomatic SCT carriers [11,12,13,14,15]. In this context, it should be considered that combined light microscopy and TEM analysis should be recommended to detect relevant microanatomical alterations. Certainly, a limitation of the study is represented by the reduced sample size. However, in our hands, the ultrastructural analysis has been instrumental to appreciate the recurrent thickening of the basement membrane at the blood–tissue interface, and to propose that a widespread endotheliopathy is likely to occur in the SCT carrier mice. Interestingly, the endothelial lining of 3 out of 5 organs (spleen, heart, and kidney) presents luminal extroflexions, highlighting the need to further investigate the possible intracellular signaling cascades leading to the formation of circulating endothelial-derived microvesicles in the SCT carriers.

## 4. Materials and Methods

### 4.1. Animal Model

The Townes mouse model was created by replacing the murine adult α-globin genes with the human α-globin gene and the murine adult β-globin genes with human sickle β^S^-globin gene fragments linked together (*HBB^S/S^:* homozygous mice harboring two SCD *HBB* alleles) [21]. Mice containing the wild-type human beta transgene were used as controls (*HBB^A/A^*: normal mice harboring two normal *HBB* alleles). Mice were purchased from The Jackson Laboratory, Bar Harbor, Maine, USA (Stock No: 013071). Heterozygous mice *(HBB^A/S^*: “sickle-cell trait” (SCT) mice harboring one normal and one SCD *HBB* allele), obtained by breeding *HBB^S/S^* with *HBB^A/A^* mice, were used as a model of SCT carrier. A total of 13 animals were used: 3 homozygotes (*HBB^S/S^*), 5 heterozygotes (*HBB^A/S^*), and 5 healthy controls (*HBB^A/A^*).

**Figure 5:**
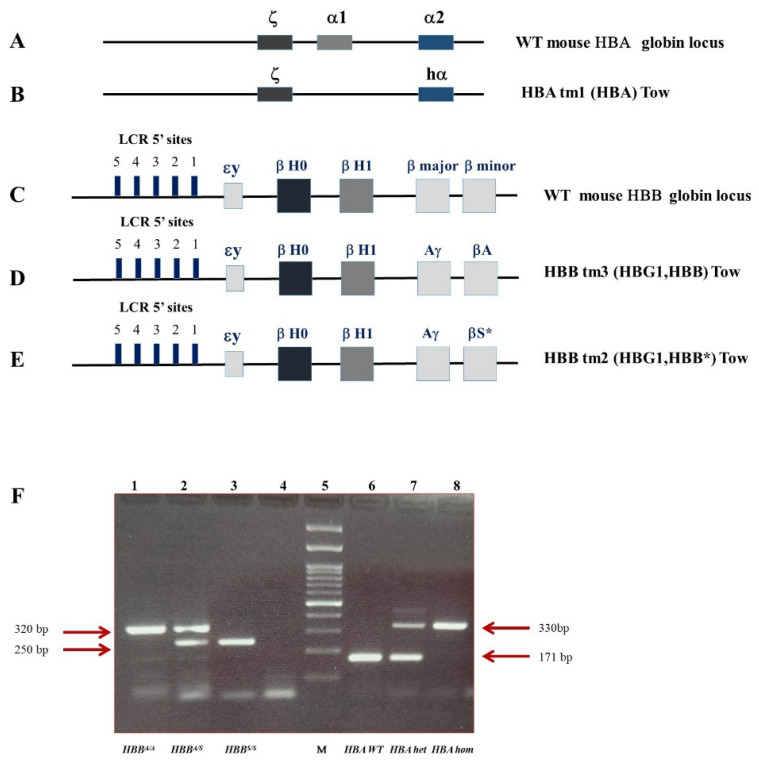
Sickle Cell Disease Humanized Mouse Model (HBA/HBS). (**A**) WT mouse HBA globin locus. (**B**) Hbatm1 (HBA) Tow mutation; designed with the human hemoglobin α gene replacing the endogenous mouse α-globin. (**C**) WT mouse HBB globin locus. (**D**) Hbbtm3 (HBG1, HBB) Tow mutation; designed with the human hemoglobin gamma (Aγ) gene and the human wt hemoglobin beta (βA) gene replacing the endogenous mouse major and minor β globin. (**E**) Hbbtm2 (HBG1, HBB*) Tow mutation; designed with the human hemoglobin gamma (Aγ) gene and the human sickle hemoglobin beta (βS) gene replacing the endogenous mouse major and minor β-globin. (**A**–**E**): Modified from Wu et al. [61] (**F**) Agarose gel electrophoresis of PCR genotyping. Lane 1: *HBB^A/A^* (320 bp); lane 2: *HBB^A/S^* (320 bp, 250 bp); lane 3 *HBB*^S/S^ (250 bp); lane 4 blank; lane 5 DNA ladder (100 bp); lane 6 WT mouse HBA (330 bp); lane 7 heterozygous HBA/HBA tm1 (330 bp, 150 bp); lane 8 homozygous HBA tm1 (170 bp).

Genotypes were determined by PCR from genomic DNA according to The Jackson Laboratory protocols and primers (Figure 5).

#### 4.1.1. Hematological Values

Peripheral blood from *HBB^A/A^*, *HBB^A/S^*, and *HBB^S/S^* anesthetized adult mice was isolated via sub-mandibular vein, collected in Microtainer ethylenediamine tetraacetic acid (EDTA) collection tubes and analyzed on a MS4 automated hematology cell counter (Melet Schloesing Laboratories, Osny, France) for hematologic values on 7 parameters (see Table 1).

#### 4.1.2. Flow Cytometry

Erythropoiesis in bone marrow cells was assayed in *HBB^A/A^* (controls), *HBB^A/S^* (SCT), and *HBB^S/S^* adult mice. Bone marrow cells suspensions were obtained, and isolated (1 × 10^6^ cells for a sample) were stained with anti-mouse Ter119 fluorescein isothiocyanate (FITC) and anti-mouse cluster of differentiation (CD)71 phycoerythrin (PE) antibodies (BD-Bioscience, San Jose, CA, USA) at a final concentration 1:100. Cells were incubated for 20 min at 4 C, washed with phosphate-buffered saline (5% bovine serum albumin), and re-suspended in fluorescence-activated cell sorting (FACS) flow solution (BD-Bioscience, San Jose, CA, USA). FACSCanto (BD-Bioscience, San Jose, CA, USA) flow cytometer was used to collect data and analyzed with FACSDiva software Version 6_1.3 (BD Biosciences, San Jose, CA, USA) and FlowJo V7_6.5. Erythropoiesis was estimated by the ratio between Ter119^+^/CD71^+^ and Ter119^+^/CD71^−^ in bone marrow cells from adult *HBB^A/A^*, *HBB^A/S^*, and *HBB^S/S^* mice. Statistical differences between means were calculated with Student’s *t*-test (see Table 2).

#### 4.1.3. Spleen Size and Organ Iron Content

Spleen size was measured as spleen index by spleen weight (mg)/body weight (g) × 100 in *HBB^A/A^*, *HBB^A/S^*, and *HBB^S/S^* mice. Liver, spleen, and heart iron content were measured as iron μg/organ in adult *HBB^A/A^*, *HBB^A/^^S^*, and *HBB^A/S^* mice. The tissue samples were shredded, incubated with acid solution for 48 h at 65C, and then combined with a chromogenic reagent. The absorbance of the solution was measured at 540 nm (Nanodrop 2000C Spectrophotometer, Thermo Fisher Scientific, Inc., Waltham, MA, USA). Statistical significance was determined for *HBB^A/S^* mice compared to *HBB^S/S^* mice and *HBB^A/A^* control mice. *p* values were calculated using a two-tailed Student’s *t*-test; *n* ≥ 3 (Table 2).

### 4.2. Histopathology

After sacrifice, liver, lung, heart, kidney, and spleen were explanted, formalin fixed for 48 h, and paraffin embedded. Four µm-thick sections, stained with Mayer’s Hematoxylin and Eosin, were used for the histopathological assessment by means of a Zeiss AxioSkope optical microscope (Carl Zeiss AG, Oberkochen, Germany). The histopathological findings were expressed as 3 degrees of injury severity for each organ (see Table 1), taking inspiration from the SCD lesion grading in the Berkeley mice reported by Manci et al. [21]. Thus, the following categories of grading were used:

- normal/minimal damage when the fields involved were from 0 to 30%
- mild/moderate damage when the fields involved were from 30 to 70%
- severe damage when the fields involved were from 70 to 100%

### 4.3. Transmission Electron Microscopy

In total, 2 healthy controls and 2 SCT carrier mice (Table 4) were analyzed by transmission electron microscopy. Immediately after surgical removal, tissue fragments of each organ were immersion fixed in 2.5% glutaraldehyde (Sigma Aldrich, St. Louis, Missouri, USA) and rapidly reduced in 1 × 1 × 3 mm frustules for TEM analysis. The tissue fixation lasted 3 h for hollow and spongy organs, and 6 h for full organs. Tissue samples were then rinsed in fresh 0.15 M phosphate buffer at 4 C. Subsequently, they were postfixed with 1% OsO_4_, dehydrated by passing them through the graded alcohol scale, and then included into epoxy resin. Semithin sections (1–2 µm), used to select the area of interest, were stained with 1% toluidine blue and observed with a Nikon (Nikon Corporation, Minato, Tokyo, Japan) inverted light microscope (mod. Ti2-U) following a validated protocol by Burattini et al. [62]. Ultrathin sections with a thickness of 50–80 nm were stained with UranyLess EM Stain (Media System Lab, Rovereto, Italy) and Reynolds’ lead citrate for the ultrastructural analysis with the Philips CM10 Electron Microscope (Philips, Amsterdam, Netherlands).

## 5. Conclusions

Our findings, in particular the signs of endothelial suffering, contribute novel data to better characterize the morpho-pathological modifications in the SCT carrier mouse and suggest the translational usefulness of this model for in-depth investigation of the subclinical multi-organ involvement detectable in patients heterozygous for HbS when subjected to environmental risk factors. It is also worth considering that elucidation of the morphological basis of SCT carriers’ lesions could provide further insight into the pathophysiology of SCD.

## Figures and Tables

**Table 1:** Hematological values for *HBB^A/A^*, *HBB^A/S^*, and *HBB^S/S^* adult mice. RBC, red blood cells; Hb, hemoglobin; Hct, hematocrit; MCV, mean corpuscular volume; MCH, mean corpuscular hemoglobin; MCHC, mean corpuscular hemoglobin concentration; RDW, red cell distribution width. All animals were analyzed at 6–12 weeks after birth; *n* ≥ 3. Data are means ± SD. *p* values were calculated by two-tailed Student *t*-test between *HBB^A/S^* and *HBB^A/A^* mice: * *p* ≤ 0.05 and between *HBB^A/S^* and *HBB^S/S^* mice: ^#^ *p* ≤ 0.05; ^##^ *p* ≤ 0.01; ^###^ *p* ≤ 0.001.

Mice	RBC (M/mm^3^)	Hb (g/dL)	Hct (%)	MCV (fl)	MCH (pg)	MCHC (g/dL)	RDW
*HBB^A/A^*	9.7 ± 0.3	12.8 ± 1.3	32.2 ± 1.7	33.2 ± 1.7	13.0 ± 0.3	39.9 ± 2.2	17.5 ± 2.8
*HBB^A/S^*	9.6 ± 0.6 ^###^	11.7 ± 0.5 ^###^	31.1 ± 1.0	32.5 ± 2.0 ^##^	12.2 ± 0.5 *	37.7 ± 1.0 ^#^	17.6 ± 2.3 ^#^
*HBB^S/S^*	6.2 ± 1.5	8.5 ± 0.8	26.3 ± 7.3	42.4 ± 4.6	15.2 ± 2.3	33.3 ± 5.0	20.6 ± 2.2

**Table 2:** Erythropoiesis in bone marrow cells, spleen size, and organ iron content assayed in adult *HBB^A/A^*, *HBB^A/S^*, and *HBB^S/S^* mice. Erythropoiesis was estimated by the ratio between Ter119^+^/CD71^+^ and Ter119^+^/CD71^−^ in bone marrow cells. Spleen size was measured as spleen index by spleen weight (mg)/body weight (g) × 100. Liver, spleen, and heart iron content were measured as iron μg/organ in adult *HBB^A/A^*, *HBB^A/S^* mice, and *HBB^S/S^* mice; *n* ≥ 3. Statistical significance was determined for *HBB^A/S^* mice compared to *HBB^A/A^* control mice and *HBB^S/S^* sickle mice. *p* values were calculated using a two-tailed Student’s *t*-test between *HBB^A/S^* and *HBB^S/S^* mice: ^##^ *p* ≤ 0.01; ^###^ *p* ≤ 0.001.

	Mean (DS):				
	Ter119^+^ CD71^+^/Ter119^+^ CD71^−^ %				
Mice	BM Cells	Spleen Index	Iron Liver, μg	Iron Spleen, μg	Iron Heart, μg
*HBB^A/A^*	1.97 ± 0.53	895 ± 272	122.57± 70.4	95.8 ± 55.6	9.3 ± 4.6
*HBB^A/S^*	2.68 ± 0.92 ^##^	1245.7 ± 203.6 ^###^	106.9 ± 30.8 ^###^	94.2 ± 28.4 ^##^	8.6 ± 4.4
*HBB^S/S^*	6.67 ± 2.03	6414 ± 684	896.4 ± 112.65	1409.76 ± 378.1	19.45 ± 8.4

**Table 3:** Semi-quantitative tissue grading (modified from Manci, et al. [21]) of the organ lesions observed in the sickle cell trait (SCT) carrier mice. n: number of examined samples.

	Liver		Spleen		Lung		Kidney		Heart	
Grade		*n*		*n*		*n*		*n*		*n*
Normal/ Minimal	minimal cell stress, preserved tissue architecture	2	minimal increase in the white pulp	0	absent to low inflammation, minimal capillary congestion	5	mononuclear infiltrates, swelling of tubular cells, vascular congestion	0	normally stained fibers, clearly visible nuclei, normal vessels, no connective fiber deposits, focal weaving	5
Mild/ moderate	mild ischemic lesion, hepatocyte vacuolization, light mononuclear infiltrates, pericentral vein tissue pallor	3	increase in white pulp, vascular ectasia	5	mononuclear infiltrates, capillary congestion, edema, dilated capillaries, thickness of intima and media of pulmonary vessels	0	mononuclear infiltrates, steatosis and swelling of tubular cells, vascular congestion	5	pale staining of myocardiocytes, ischemic swelling, disorganization of histoarchitecture	0
Severe	large areas of necrosis, infarcts, mononuclear infiltrates, endovascular sickle cells	0	gross infarcts, increased spleen diameter, pigment deposits	0	infarcts, emphysema, hemorrhage, edema, thickness of intima and media of pulmonary vessels	0	gross infarcts, glomerular necrosis, papillary necrosis	0	Fragmentation and pale staining of myocardiocytes, nuclei not clearly visible, large areas of coagulative necrosis	0

**Table 4:** Sickle Cell Trait carrier mice (M1 and M2) and controls (C1 and C2) used for the TEM multi-organ analysis.

	Genotype	Sex
Control 1 (C1)	*HBB^A/A^*	Male
Control 2 (C2)	*HBB^A/A^*	Male
Mouse 1 (M1)	*HBB^A/S^*	Male
Mouse 2 (M2)	*HBB^A/S^*	Male

## Data Availability

The data presented in the current study are available from the corresponding author on reasonable request.
